# Perceptions of fatigue and neuromuscular measures of performance fatigability during prolonged low‐intensity elbow flexions

**DOI:** 10.1113/EP090981

**Published:** 2023-02-10

**Authors:** Monica Marzouk, Daniel J. McKeown, David N. Borg, Jonathon Headrick, Justin J. Kavanagh

**Affiliations:** ^1^ Neural Control of Movement Laboratory Menzies Health Institute Queensland Griffith University Gold Coast Queensland Australia; ^2^ The Australian Centre for Health Services Innovation and Centre for Healthcare Transformation, School of Public Health and Social Work Queensland University of Technology Brisbane Queensland Australia

**Keywords:** electromyography, fatigue, predictive modelling, transcranial magnetic stimulation

## Abstract

The purpose of this study was to determine the relationship between transcranial magnetic stimulation (TMS) measures of performance fatigability and commonly used scales that quantify perceptions of fatigue during exercise. Twenty healthy participants (age 23 ± 3 years, 10 female) performed 10 submaximal isometric elbow flexions at 20% maximal voluntary contraction (MVC) for 2 min, separated by 45 s of rest. Biceps brachii muscle electromyography and elbow flexion torque responses to single‐pulse TMS were obtained at the end of each contraction to assess central factors of performance fatigability. A rating of perceived exertion (RPE) scale, Omnibus Resistance scale, Likert scale, Rating of Fatigue scale and a visual analogue scale (VAS) were used to assess perceptions of fatigue at the end of each contraction. The RPE (root mean square error (RMSE) = 0.144) and Rating of Fatigue (RMSE = 0.145) scales were the best predictors of decline in MVC torque, whereas the Likert (RMSE= 0.266) and RPE (RMSE= 0.268) scales were the best predictors of electromyographic amplitude. Although the Likert (RMSE = 7.6) and Rating of Fatigue (RMSE = 7.6) scales were the best predictors of voluntary muscle activation of any scale, the number of contractions performed during the protocol was a better predictor (RMSE = 7.3). The ability of the scales to predict TMS measures of performance fatigability were in general similar. Interestingly, the number of contractions performed was a better predictor of TMS measures than the scales themselves.

## Abstract (highlights)

- **What is the central question of this study?**
  What is the predictive relationship between self‐reported scales to quantify perceptions of fatigue during exercise and gold standard measures used to quantify the development of neuromuscular fatigue?
- **What is the main finding and its importance?**
  No scale was determined to be substantively more effective than another. However, the number of ongoing contractions performed was shown to be a better predictor of fatigue in the motor system than any of the subjective scales.

## 1 INTRODUCTION

During prolonged submaximal exercise, declines in the motor system can occur that may impact the ability to perform the task (Kluger et al., 2013). This is often reflected by increases in performance fatigability (progressive reduction in muscle activation and maximal force; Amann et al., 2006; Bigland‐Ritchie et al., 1986; Sogaard et al., 2006) and perceptions of fatigue (increased subjective sensations of effort, exertion and muscle fatigue; Borg, 1990; Marcora, 2009). While there are several neurophysiological techniques capable of quantifying performance fatigability, researchers commonly include self‐reported scales to capture an individual's perceptions of fatigue. Several studies have examined the relationship between perceptions of fatigue and maximal voluntary contraction (MVC) force (Buckley & Borg, 2011), muscle contractile function (Abdel‐Malek et al., 2020) and walking ability (Galloway et al., 2022), to name a few. However, the relationship between perceived fatigability and the ability of the central nervous system (CNS) to activate muscle remains unclear.

Self‐reported scales have been developed to quantify perceptions of fatigue during exercise (Leung et al., 2004; Micklewright et al., 2017; Robertson et al., 2003). These scales are used for a variety of exercise modalities, with only a few having a physiological foundation in their development. In particular, the Borg rating of perceived exertion (RPE) scale has a linear correlation with heart rate and exercise intensity during aerobic activity (Borg, 1982), while a simple ‘fatigue score’ created with a visual analogue scale (VAS) has a near‐linear relationship with the intensity and duration of muscle activation (Leung et al., 2004; Lyons et al., 1993). Of the few studies that have assessed relationships between self‐reported scales on perceptions of fatigue and the ability of the CNS to activate muscle, the focus has been on changes in the myoelectric domain. For example, the relationship between the RPE scale and electromyographic (EMG) activity (amplitude and mean power frequency) for muscle is low during weak isometric contractions (Chan et al., 2000), but increases during strong and repeated muscle contractions (Hummel et al., 2005; Kankaanpaa et al., 1997; Oberg et al., 1994). Therefore, it is possible that some self‐reported scales demonstrate clearer alignment with muscle activation compared to others. Given that no gold standard measure of perceptions of fatigue has been agreed upon, it is of interest to determine how these scales align with well‐established quantitative measures of voluntary muscle activation (VA). Assessing this relationship is especially pertinent for applications where quantitative measures of VA are not feasible (e.g., athletic training) and scales are used as a ‘proxy’ to ascertain perceptions of fatigue across various training modalities. By determining the strength of relationship between a subjective scale(s) and a measure of performance fatigability, researchers and practitioners can effectively evaluate the use of scales in experimental/training designs to supplement objective quantitative measures most effectively.

Transcranial magnetic stimulation (TMS) has proven to be a valuable tool in assessing excitability in corticospinal pathways, as well as the ability of the CNS to drive a muscle to create force. A magnetic stimulus to the motor cortex elicits twitch responses in a target muscle and is used to determine if voluntary output from the motor cortex is submaximal during brief MVCs (Gandevia et al., 1996). Comparing the superimposed twitch obtained from supramaximal stimulation during isometric MVCs to the size of the resting twitch estimated from linear regression of responses obtained at submaximal intensities (above 50% MVC) provides a measure of VA (Todd et al., 2003). As VA reflects the level of descending neural drive to a muscle, it represents the reduced ability of the CNS to activate muscle during exercise (Merton, 1954). Motor evoked potentials (MEP) recorded with electromyography reflect corticospinal excitability, where the amplitude and area of the MEP progressively increases in line with decreases in voluntary drive to the muscle during sustained contractions (Sacco et al., 1997; Taylor et al., 1996). The MEP is followed by a period of cortical‐ and spinal‐derived inhibition that results in temporary cessation of EMG activity. This silent period indicates the level of GABAergic inhibition imposed on cortical and spinal motoneurones (Ziemann et al., 1995) and changes within the inhibitory hyperdirect pathway of the basal ganglia (Priori et al., 1994). During submaximal contractions, silent period duration increases as neural networks involved in inhibition increase in activity (Taylor et al., 2000).

In the current study, we assessed the relationship between TMS measures of corticospinal excitability and VA, and self‐reported perceptions of fatigue during a repetitive sustained submaximal elbow flexion protocol. For clarity, definitions of fatigue are aligned with the taxonomy proposed by Kluger et al. (2013), where performance fatigability is defined as ‘changes in corticospinal excitability and VA associated with fatigability during motor tasks’, and perceptions of fatigue are defined as ‘psychological factors that contribute to the perception of fatigability when performing motor tasks’. It was hypothesised that performance fatigability would progressively increase during the sustained submaximal contraction protocol, which would be reflected in reductions in MVC and VA and increases in all self‐reported scales on perceptions of fatigue. It was also hypothesised that scales assessing perceptions of fatigue that have a physiological underpinning during sustained exercise, would best predict with TMS measures.

## 2 METHODS

### 2.1 Ethical approval

Approval for testing procedures was obtained from the Human Research Ethics Committee at Griffith University (Ref: 2020/785). Written informed consent was obtained from all participants prior to testing. All procedures were performed in accordance with the *Declaration of Helsinki*, except for registration in a database.

### 2.2 Participants

Twenty healthy recreationally active participants volunteered to participate in this study (age 23 ± 3 years, 10 female). Each participant was screened using a medical history questionnaire, which contained exclusion criteria specific to neurological and musculoskeletal injury, TMS, electrical nerve stimulation, and prescription medications such as antidepressants and anxiolytics. Participants were instructed to refrain from any stimulants or depressants such as caffeine, alcohol or exercise for at least 8 h before testing. Power calculations were performed with G*Power software (v3.1.9.4). To achieve a statistical power of 0.8 at an α‐level of 0.05 and to determine a large effect, it was determined that at least 19 participants were required for this study.

### 2.3 Electromyography and torque measurements

**FIGURE 1:**
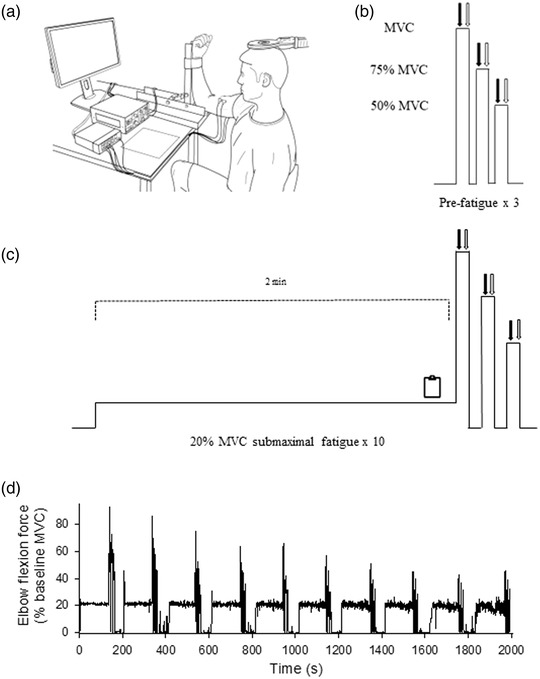
(a) Elbow flexion force was measured by attaching the participant's forearm to a custom‐designed transducer. (b) Pre‐fatigue measurements consisted of three ramped contraction (maximal voluntary contraction (MVC), 75% MVC and 50% MVC. The peak MVC was used to calculate the 20% MVC sustained contraction for the fatigue protocol. (c) To induce fatigue, participants were required to sustain a 20% MVC for 2 min, which was immediately followed by a MVC, 75% MVC and 50% MVC. During the last 20 s of the sustained contraction, self‐reported scales were employed to assess perceptions of fatigue. (d) During the MVC, 75% MVC and 50% MVC, transcranial magnetic stimulation (TMS) and brachial plexus stimulations was applied to elicit twitch and electromyography (EMG) responses in the elbow flexor muscles. This sustained contraction protocol was repeated 10 times with 45 s rest between each attempt (representative trace from participant). Filled arrows (b, c) indicate TMS, open arrows indicate brachial plexus stimulation, and clipboard (c) indicates timing of self‐reported scales.

Participants were positioned in a chair with their right arm fixed in a custom‐built transducer to measure isometric elbow flexions (Figure 1a). The shoulder and elbow were placed in 90 degrees of flexion and the participant's arm was fixed to the device at the wrist using a non‐compliant strap. A precision S‐beam load cell (PT4000, PT Ltd, Auckland, New Zealand) with a 1.1 kN range and full‐scale output of 3 mV/V was used to measure elbow flexion force, which was converted to elbow flexion torque. To provide real‐time feedback on torque generation, a computer monitor was positioned ∼1 m in front of participants at eye level. Horizontal target lines were presented on the screen to aid in tracking of the contraction protocol. Surface EMG was recorded from the biceps brachii muscle and triceps brachii muscle. Bipolar Ag–AgCl electrodes (24 mm diameter, Kendall Arbo, Cardinal Health Inc., Dublin, OH, USA) were attached to the skin over each muscle with a 24 mm inter‐electrode distance. A ground electrode was placed on the acromion of the test limb. Force and EMG signals were sampled at 2000 Hz using a 16‐bit analog‐to‐digital converter (CED 1401; Cambridge Electronic Design, Cambridge, UK) and Spike2 software (version 7.02; Cambridge Electronic Design). EMG signals were amplified (×300) and bandpass filtered (10–1000 Hz) using a CED 1902 amplifier (Cambridge Electronic Design) whereas force signals remained unfiltered.

### 2.4 Brachial plexus stimulation

Using a constant current stimulator (DS7AH, Digitimer Ltd, Welwyn Garden City, UK), single electrical pulses of 100 μs duration were delivered to the brachial plexus to measure the maximum compound action potential (*M*_max_) of the biceps brachii and triceps brachii muscles. A surface anode was positioned over the ipsilateral acromion and a surface cathode was positioned over the supraclavicular fossa. Optimal positioning of the cathode was determined by using a motor point stimulating pen before affixing the surface electrode. To determine the optimal stimulus intensity, the stimulator current was progressively increased until the *M*_max_ of both the biceps brachii muscle and triceps brachii muscle was reached. The stimulus intensity for experimental testing was then set at 130% of *M*_max_ (52–234 mA).

### 2.5 Cortical stimulation

MEPs were elicited in the biceps brachii muscle using a 90 mm circular stimulating coil attached to a Magstim 200^2^ transcranial magnetic stimulator (Magstim Co., Whitland, UK). The coil was positioned over the vertex and oriented to preferentially activate the left motor cortex, which was maintained for the duration of the experiment. The stimulator intensity which elicited the highest elbow flexor twitch torque with a MEP that was greater than 80% of the biceps brachii *M*_max_ and less than 20% of the triceps brachii *M*_max_ during a brief isometric contraction at 50% MVC was selected as the optimal stimulus intensity for each testing session (70–75% stimulus output). This criterion ensured that the TMS pulse activated a large proportion of the biceps brachii motoneuron pool whilst minimising activation of the triceps brachii in all participants.

### 2.6 Torque and EMG‐derived measures

Maximal voluntary torque was calculated from MVCs using a 50 ms window preceding the stimulation artefact. Decline in MVC throughout the contraction protocol was indicative of performance fatigability, where the maximal force generating capacity of the elbow flexors was compromised by the low‐intensity sustained contractions. Force and EMG responses to TMS were used to determine central factors contributing to performance fatigability. Superimposed twitch amplitude was calculated from the evoked changes in torque during motor cortical stimulation. Resting twitch torques were estimated from the superimposed twitch amplitudes during the ongoing brief MVC, and 75% MVC and 50% MVC performed at baseline and at the end of each 2 min low‐intensity sustained contraction. A linear regression calculated the *y*‐intercept between the amplitude of the superimposed twitch and voluntary torque. The linear regression was performed for each maximal effort, 75% MVC and 50% MVC associated with the same contraction block. The level of VA was calculated using the equation: VA (%) = (1 − superimposed twitch/resting twitch) × 100 (Todd et al., 2003). MEP area was measured from each TMS and then normalised to the area of the *M*_max_ for the same contraction to account for activity‐dependent changes in muscle fibre action potentials. The silent period duration was the time from the delivery of the TMS pulse (EMG stimulus artefact) to the continuous recommencement of voluntary EMG activity and was determined by visual inspection.

### 2.7 Self‐reported scales

Perceptions of fatigue were assessed using five self‐reported scales administered during each contraction block. These scales were selected after performing a comprehensive review of the literature and were identified as scales that were commonly used in exercise testing. These scales consisted of an RPE scale (Noble et al., 1983), Omnibus Resistance (OMNI) scale (Lagally & Robertson, 2006), Likert scale (Grant et al., 1999), Rating of Fatigue scale (Micklewright et al., 2017) and a VAS (Grant et al., 1999). Participants were familiarised to the scales and how to respond to them the day before testing. Scales were completed at the end of each 20% MVC contraction but before the onset of the MVCs. The five self‐reported scales were presented in the final 20s of the 20% MVC contraction and completed in a randomised order between participants. The specifics of the self‐reported scales were:

1. The RPE scale was presented as a table where numerical values were matched to descriptive text. These were: 1, ‘Nothing’; 2, ‘Very easy’; 3, ‘Easy’; 4, ‘Comfortable’; 5, ‘Somewhat difficult’; 6, ‘Difficult’; 7, ‘Hard’; 8, ‘Very hard’; 9, ‘Extremely hard’; 10, ‘Maximal/exhaustion’. Participants were required to verbally indicate their score on the RPE scale.
2. The OMNI scale was presented as a 20 cm line with equally spaced ticks. The ticks corresponded to the labels: 0, ‘Extremely easy’; 1, 2, ‘Easy’; 3, 4, ‘Somewhat easy’; 5, 6, ‘Somewhat hard’, 7, 8, ‘Hard’; 9, 10, ‘Extremely hard’. At four locations throughout the OMNI scale a visual of an exercising human was presented, where a barbell was lifted above the head with increasing number of weights on the ends of the bar. Participants were required to verbally indicate their score on the OMNI scale.
3. The Likert scale measured participant level of agreement to the written statement ‘I am experiencing fatigue’. The scale presented: 1, ‘Strongly agree’; 2, ‘Agree’; 3, ‘More or less agree’; 4, ‘Undecided’; 5, ‘More or less disagree’; 6, ‘Disagree’; 7, ‘Strongly disagree’. The direction of this scale was the reverse of other scales in the study, whereby a higher value corresponded to lesser subjective sensation of fatigue. Participants were required to verbally indicate their score on the Likert scale.
4. The Rating of Fatigue scale was presented across 20 cm as a series of equally spaced numbers ranging from 0 to 10. A value of 0 was accompanied by the text ‘Not fatigued at all’ and the value of 10 was accompanied by ‘Total fatigue and exhaustion’. Participants were required to verbally indicate their score on the Rating of Fatigue scale.
5. The VAS was a 20 cm line without predefined anchors or units. The only text presented on the scale was at the origin of the line which was labelled ‘No fatigue’ and at the end of the line which was labelled ‘Severe fatigue’. Participants were asked to mark a point along the line that they believed represented their subjective sensation of fatigue.

### 2.8 Experiment protocol

Prior to performing the sustained contractions, each participant performed 3–5 brief (∼2 s) maximal effort elbow flexions to establish a baseline MVC (Figure 1b). The maximal effort contraction that had the highest torque output was considered as the participant's MVC. Once MVC was determined, participant performed 75% MVCs and 50% MVCs, each of which was separated by 3 s of rest. During all baseline contractions a cortical stimulation and a brachial plexus stimulation was delivered to the participant with a 2 s interstimulus interval to establish baseline responses. Strong verbal encouragement was present during all maximal efforts. The trial with the highest peak force was used as the participant's MVC. The contraction protocol consisted of 10 contraction blocks. Each contraction block was an isometric 20% MVC elbow flexion that was held for 2 min. Following each 2 min, the participant immediately performed an MVC, a 75% MVC and a 50% MVC, each of which was separated by 3 s of rest (Figure 1c). During each of these graded contractions, a cortical stimulation and a brachial plexus stimulation was delivered.

### 2.9 Data and statistical analysis

All analyses were undertaken in R. Linear mixed‐effect models were used to determine changes in force and TMS measures across the exercise task (for exploratory plots see Supplement 1 at Figshare; https://doi.org/10.6084/m9.figshare.19775755). Models included contraction block as a fixed effect—as a B‐spline (five evenly spaced knots), second order polynomial, or linear term—and a random intercept for each participant in the study. Contrasts between the end of each contraction block and baseline were made using the package *emmeans* (Lenth et al., 2021) with *P*‐values corrected for 10 comparisons. Hedges’ *g* was calculated for the standardised difference. Ordinal regression was used to determine if the RPE scale, OMNI scale, Likert scale, and Rating of Fatigue scale responses changed across the exercise task. The RPE scale, OMNI scale, and Rating of Fatigue scale model included time as a fixed effect and a random intercept for each participant. The Likert scale model included time as a fixed effect. Linear regression was used to model VAS responses, with a non‐linear term for block (second order polynomial) included as a fixed effect, and a random intercept for each participant.

Linear mixed‐effect models were used to determine which scale best predicted MVC torque, VA, estimated resting twitch torque, EMG root mean square (RMS) amplitude, normalized MEP area, and silent period duration. Five models were fit to each outcome, with the RPE scale, OMNI scale, Likert scale, Rating of Fatigue scale or VAS included as a predictor variable in each model (fixed effect). Because the RPE scale, OMNI scale, Likert scale and Rating of Fatigue scale are ordinal variables, they were coded as factors, with 10, 9, 10 and 7 levels, respectively. The VAS was included as a continuous predictor and fit as a B‐spline (five evenly spaced knots), second order polynomial, or linear term. All models included a random intercept for each participant. To identify the best fitting model, the five models, and a base model including contraction number, were ranked based on the root‐mean‐square error (RMSE) statistic. The RMSE is the SD of the residuals (i.e., prediction errors), with lower values indicating a better performing model and a scale that can predict a torque and EMG‐derived measure better over another scale with a higher RMSE. Cross validation (10‐fold, with five repeats) was used to estimate the average RMSE for each model, with folds balanced for participants (for explanation, see Supplement 2 at Figshare; https://doi.org/10.6084/m9.figshare.19775755). RMSE values were used to identify the best fitting model, with lower values indicting better predictive performance. Although marginal and conditional coefficients of determination (R^2^) were also obtained across model fits from the cross‐validation procedure, they were not used for model selection (for explanation see Supplement 2 at Figshare; https://doi.org/10.6084/m9.figshare.19775755). Information criterion (e.g., Akaike's or Bayesian) indices were not used for model selection, to avoid penalising models for the number of parameters, which reflect the number of levels on a given scale. Finally, the predicted values from the best fitting model(s) were visually inspected to determine whether there was a monotonic change in the outcome variable, across the levels of the scale, that is, whether increases in scale ratings were associated with increases (or no change) in the outcome variable of interest.

Data are reported as the estimated marginal mean and 95% confidence interval (CI), unless otherwise stated. The α‐level for all tests was set at 5%. All linear mixed‐effect models were fit using the *lmerTest* package (Kuznetsova et al., 2017), and ordinal regression models using the *ordinal* package. There was 0.45% missing data for normalized MEP area. No values were imputed for the missing observations. The R code used to produce the analyses is available at https://github.com/SciBorgo/subjective‐scales‐neuro.

## 3 RESULTS

### 3.1 Torque‐ and EMG‐derived measures during the contraction protocol

**FIGURE 2:**
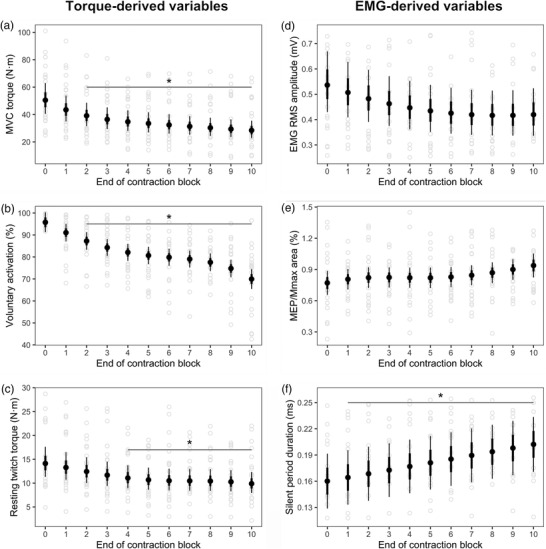
Performance fatigability throughout the contraction protocol. Data in the left column represent the torque‐derived variables of MVC torque (a), voluntary activation (b), and estimated resting twitch amplitude (c), presented across the 10 contraction blocks performed by participants. Data in the right column represent EMG‐derived variables of RMS amplitude (d), normalized MEP area (e), and silent period duration (f) of the biceps brachii muscle. All measurements were obtained at the completion of each 2 min submaximal contraction. The contraction block labelled 0 is the baseline measurement for the variable. Data are presented as the marginal mean, and error bars indicate the 68% (thick inner line) and 95% (thin line) confidence intervals. Light grey open circles indicate individual participant data. Asterisk indicates statistically different from baseline measures. MVC torque (*P* < 0.0001–0.010) and voluntary activation (*P* < 0.0001–0.029) were lower from the end of contraction block 2 onwards. Resting twitch torque was lower from the end of block 4 onwards (*P* = 0.001–0.017). Silent period duration was longer from the end of contraction block 1 onwards (*P* = 0.005–0.007). *n* = 20 participants.

All participants were able to successfully complete the contraction protocol and TMS procedures in this study. The performance of each contraction block generated substantial fatigue, as the MVC progressively declined from the first block to the tenth block. There was an effect of contraction block on MVC torque, VA, estimated resting twitch torque, EMG RMS amplitude, and silent period duration. MVC torque (*P* < 0.0001–0.010; *g* = −1.06 to −0.57) and VA (*P* < 0.0001–0.029; *g* = −2.78 to −1.00) were lower from contraction block 2 onwards (Figure 2a and b). Resting twitch torque was lower from block 4 onwards (*P* = 0.001–0.017; *g* = −0.75 to −0.52, Figure 2c). After correcting for multiple testing, EMG RMS amplitude was not statistically different at any point between contraction block 1 and 10 compared to baseline (*P* = 0.132–1; *g* = 0.14–0.55, Figure 2d). Normalized MEP area was not statistically different at any point between contraction block 1 and 10 compared to baseline (*P* = 0.952–1; *g* = 0.01–0.52, Figure 2e). Silent period duration was longer from contraction block 1 onwards (*P* = 0.005–0.007; *g* = 0.06–0.56, Figure 2f).

### 3.2 Self‐reported scales during the contraction protocol

**FIGURE 3:**
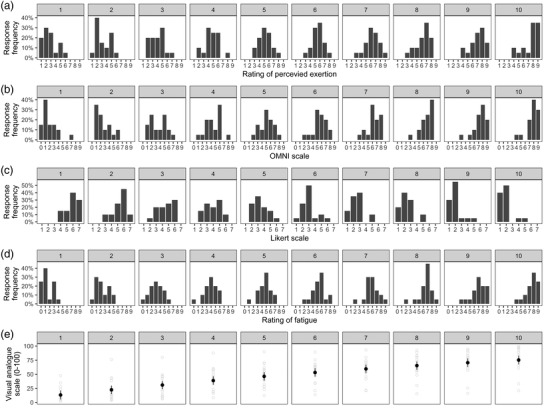
Rating of perceived exertion (RPE; a), Omnibus (OMNI) Resistance scale (b), Likert scale (c), Rating of Fatigue (d), and visual analogue scale (e) responses across the exercise task. Responses in (a–d) are reported as the frequency, with values in (e) reported as the mean and 95% confidence interval. Light grey open circles in (e) indicate individual participant data (*n* = 20 participants).

There was an effect of contraction block on all scales. The RPE scale (odds ratio (OR) = 1.36, 95% CI: 1.14–1.57, *P* < 0.0001; Figure 3a), OMNI scale (OR = 1.64, 95% CI: 1.38–1.90, *P* < 0.0001; Figure 3b), Rating of Fatigue scale (OR = 1.86, 95% CI: 1.57–2.15, *P* < 0.0001; Figure 3d) and VAS (all *P*‐values <0.0001, *g* = 0.27–3.41; Figure 3e) responses increased with each contraction block. Ratings on the Likert scale decreased with each contraction block (OR = 0.48, 95% CI: 0.42–0.55; *P* < 0.0001; Figure 3c). Boxplots of the RPE, OMNI, Rating of Fatigue and Likert scale responses across the contraction blocks are shown in Supplement 3 at Figshare (https://doi.org/10.6084/m9.figshare.19775755).

### 3.3 Predictive model performance

**FIGURE 4:**
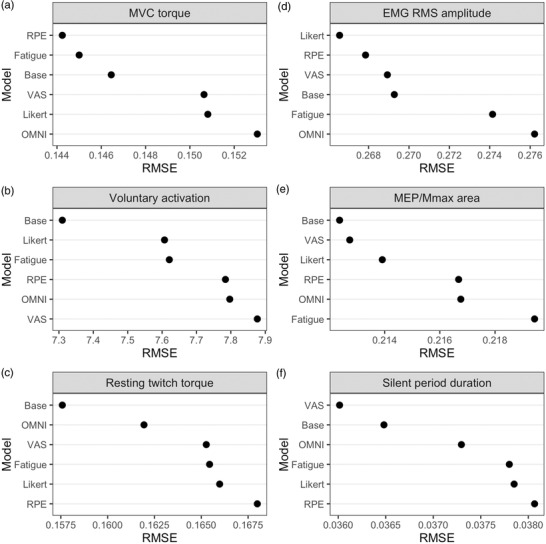
Root‐mean‐square error (RMSE) of models predicting maximal voluntary contraction (MVC) torque (a), voluntary activation (b), estimated resting twitch torque (c), EMG RME amplitude (d), MEP/*M*_max_ area (e), and silent period duration (f). MVC torque, estimated resting twitch torque and EMG RMS amplitude were logged before analysis. Models are ranked according to their RMSE value, in descending order, with smaller RMSE values indicating a better performing model. Base, a base model where the number of the contraction (contraction block) is included as a predictor variable; RPE, rating of percevied exertion; Fatigue: rating of fatigue; VAS: visual analogue scale.

**FIGURE 5:**
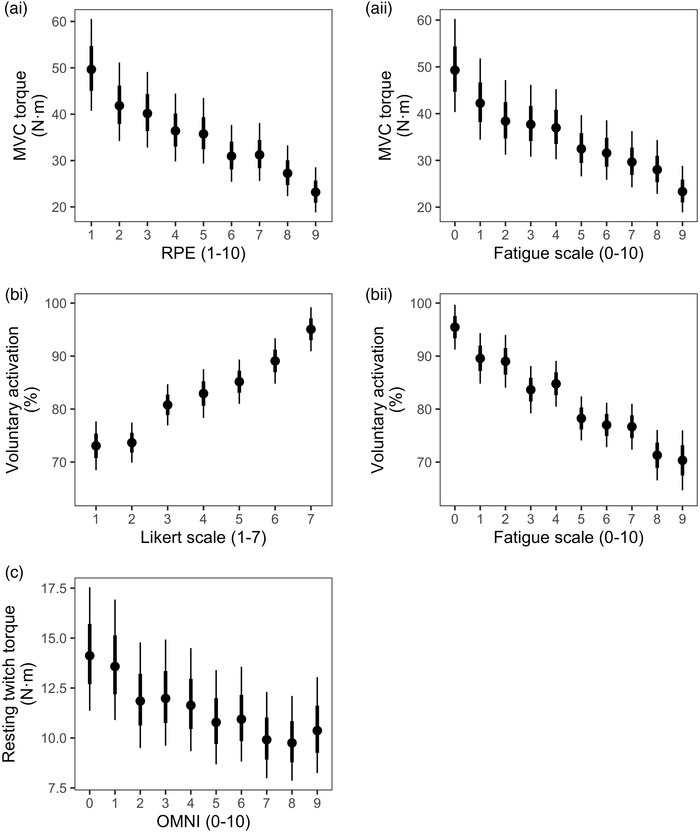
Fitted values from the models that best predicted maximal voluntary contraction (MVC) torque (a), voluntary activation (b), and estimated resting twitch torque (c). When two models predicted similarly well, based on their root‐mean‐square error statistic, both models are shown (i.e., MVC torque and voluntary activation). Data are presented as the marginal mean and error bars indicate the 68% (thick inner line) and 95% (thin line) confidence interval.

The RPE scale was the best predictor of MVC torque, marginally, followed closely by the Rating of Fatigue scale (Figure 4a). Ratings across the RPE and Rating of Fatigue scale were generally able to differentiate between MVC torque values (Figure 5ai and ii). After the base model, the Likert and Rating of Fatigue scales were marginally the best predictors of VA (Figure 4b). Ratings on the Likert scale were generally able to differentiate between VA values (Figure 5bi), more so than the Rating of Fatigue scale (Figure 5b ii). After the base model, the OMNI scale was marginally the best predictor of estimated resting twitch torque (Figure 4c). Ratings on the OMNI scale was generally poor at distinguishing between different estimated resting twitch torque values (Figure 5c).

**FIGURE 6:**
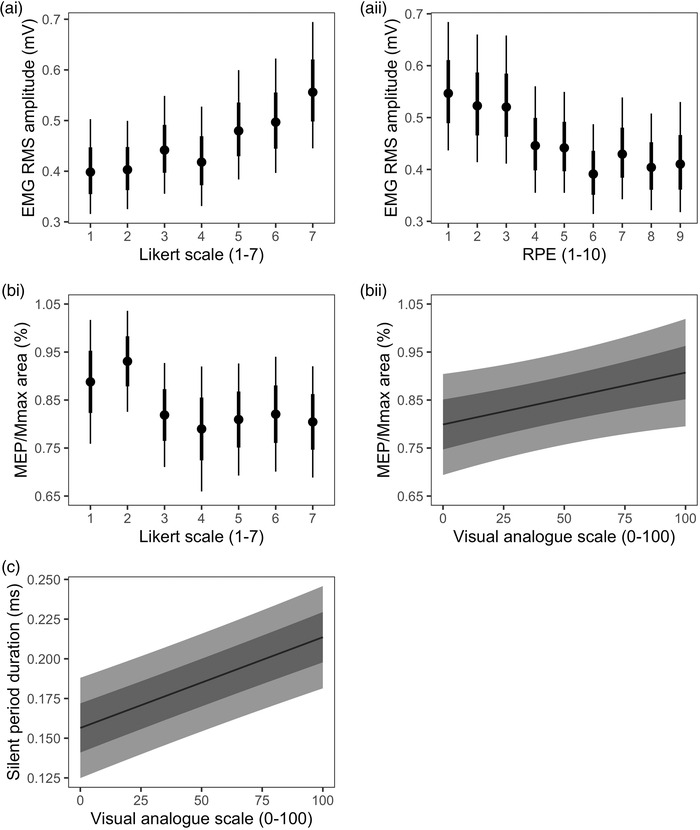
Fitted values from the models best predicting EMG RMS amplitude (a), MEP/*M*_max_ area (b), and silent period duration (c). When two models predicted similarly well, based on their root‐mean‐square error statistic, both models were shown (i.e., EMG RMS amplitude and MEP/*M*_max_ area). Data are presented as the marginal mean and error bars indicate the 68% (thick inner line, or darker inner grey ribbon) and 95% (thin line, or outer lighter grey ribbon) confidence intervals.

The Likert scale was marginally the best predictor of EMG RMS amplitude, followed closely by the RPE scale (Figure 4d). Ratings in the lower and upper levels of both scales were generally poor at distinguishing between different EMG RMS amplitude values (Figure 6ai and ii). After the base model, the Likert scale and VAS were marginally the best predictors of normalized MEP area (Figure 4e). Ratings 3−7 on the Likert scale were not able to distinguish between different normalized MEP area values (Figure 6bi), while higher VAS ratings associated with higher normalized MEP area values (β = 0.001, 95% CI: 0.00001–0.002; Figure 6b ii). The VAS was marginally the best predictor of the silent period duration (Figure 4f), with higher ratings associated with longer silent period durations (β = 0.001 s, 95% CI: 0.004–0.008; Figure 6c).

The marginal and conditional *R* ^2^ for each model is reported in Table 1 for torque‐derived variables, and Table 2 for EMG‐derived variables. All fitted models explained ∼90% of the variation in MVC torque and estimated resting twitch torque, ∼75% of the variation in EMG RMS amplitude and silent period duration, ∼65% of the variation in VA, and ∼50% of the variation in normalized MEP area.

**TABLE 1:** Marginal and conditional coefficient of determination (*R* ^2^) for models predicting maximal voluntary contraction torque, voluntary activation and estimated resting twitch torque.

Model	Marginal *R* ^2^	Conditional *R* ^2^
Maximal voluntary contraction torque		
Base model	0.110	0.924
Rating of fatigue	0.162	0.920
Likert	0.140	0.911
OMNI	0.152	0.910
Rating of perceived exertion	0.169	0.919
Visual analogue scale	0.153	0.908
Voluntary activation		
Base model	0.320	0.688
Rating of fatigue	0.361	0.671
Likert	0.351	0.658
OMNI	0.330	0.669
Rating of perceived exertion	0.350	0.670
Visual analogue scale	0.321	0.638
Resting twitch torque		
Base model	0.051	0.906
Rating of fatigue	0.057	0.900
Likert	0.050	0.898
OMNI	0.062	0.902
Rating of perceived exertion	0.055	0.897
Visual analogue scale	0.050	0.894

The base model included *contraction block* as a fixed effect. Maximal voluntary contraction torque and estimated resting twitch torque were logged before analysis. The marginal *R* ^2^ reflects the variance in the outcome variable explained by the fixed effects in the model only, and the conditional *R* ^2^ refelects the variance explained by both the fixed and random effects. All models included a random intercept for each participant in the study. *n* = 20 participants. OMNI, Omnibus Resistance scale.

**TABLE 2:** Marginal and conditional coefficient of determination (*R* ^2^) for models predicting EMG RMS amplitude, MEP/*M*_max_ area and silent period duration.

Model	Marginal *R* ^2^	Conditional *R* ^2^
EMG RMS amplitude		
Base model	0.026	0.749
Rating of fatigue	0.046	0.748
Likert	0.047	0.757
OMNI	0.041	0.749
Rating of perceived exertion	0.050	0.757
Visual analogue scale	0.033	0.745
MEP/*M*_max_ area		
Base model	0.024	0.492
Rating of fatigue	0.025	0.486
Likert	0.037	0.479
OMNI	0.033	0.492
Rating of perceived exertion	0.031	0.502
Visual analogue scale	0.013	0.478
Silent period duration		
Base model	0.032	0.776
Rating of fatigue	0.048	0.769
Likert	0.037	0.766
OMNI	0.052	0.780
Rating of perceived exertion	0.046	0.770
Visual analogue scale	0.048	0.785

The base model included *contraction block* as a fixed effect. EMG RMS amplitude was logged before analysis. The marginal *R* ^2^ reflects the variance in the outcome variable explained by the fixed effects in the model only, and the conditional *R* ^2^ refelects the variance explained by both the fixed and random effects. All models included a random intercept for each participant in the study. *n* = 20 participants. OMNI, Omnibus Resistance scale.

## 4 DISCUSSION

This study assessed the relationship between TMS measures of corticospinal excitability and VA, and self‐reported perceptions of fatigue during a repetitive sustained submaximal elbow flexion protocol. First, changes in ratings from five self‐reported scales and TMS measures across the contraction protocol were quantified. Second, five models were fitted to the data where each model included a different self‐reported scale as a predictor variable. The five models were ranked according to their predictive performance to identify the best fitting model. The main findings of this study were: (1) the contraction protocol caused progressive changes in TMS‐related measures associated with performance fatigability and self‐reported perceptions of fatigue; (2) although the scales were unable to outperform each other in predicting the TMS measures associated with performance fatigability, the number of contractions performed was the better predictor.

### 4.1 The elbow flexion protocol caused progressive declines in performance

Impairments in both the CNS and muscle have been shown to progressively increase during sustained submaximal contractions, which has the effect of reducing maximal force generating capacity (Sogaard et al., 2006). In the current study VA progressively decreased, which suggests that the contraction protocol reduced output from the motor cortex, or reduced responsiveness of α‐motoneurones to descending drive (Finn et al., 2018; Smith et al., 2007). However, as the estimated resting twitch progressively decreased, peripheral factors also contributed to the MVC impairment. Indeed, changes in the contractility of muscle fibres is commonly reported with sustained contraction protocols (Allen et al., 2002; Burnley et al., 2012). Our findings support the viewpoint that no single neuromuscular component can explain the development of performance fatigability during a prolonged contraction protocol (Carpentier et al., 2001; Farina et al., 2009; Martin et al., 2008). Moreover, our findings allow us to explore (with predictive modelling) if decrements in the motor system align with concurrently measured perceptions of fatigue. With respect to EMG responses in the current study, the silent period duration progressively lengthened throughout the contraction protocol. Lengthening of the silent period is routinely reported during prolonged elbow flexions (Taylor et al., 2000; Taylor et al., 1996; Thorstensen et al., 2020), and signifies a greater degree of α‐motoneuronal refractiveness and/or reduced excitability of the motor cortex (Taylor et al., 1996). The underlying cortical mechanism is likely due to enhanced GABAergic inhibition onto motoneurones (Ziemann et al., 1995). The findings of the current study suggest that these central factors (insufficient voluntary drive to spinal motoneurones and increased cortical inhibition) were associated with the development of performance fatigability during the repeated submaximal contractions.

### 4.2 The elbow flexion protocol caused progressive increases in perceptions of fatigue

Perceptions of fatigue are expected to change concurrently with changes in performance fatigability throughout a submaximal sustained contraction. This is due to corollary discharge of corticofugal motor commands onto somatosensory cortices (subjective sensations of effort; Aniss et al., 1988; Enoka & Stuart, 1992), as well as feedback from metabosensitive afferents in the periphery (subjective sensations of exertion; Brooks et al., 2013; Pageaux et al., 2015). This explains why increases in the perceptions of fatigue are commonly reported during prolonged muscle activation (Amann et al., 2011), in environments where substantial deoxygenation of the cortex occurs (McKeown et al., 2020) and in individuals who are characterised by pathological fatigue of central origin (i.e., multiple sclerosis and chronic fatigue syndrome; Sacco et al., 1999; Thickbroom et al., 2006).

In the current study, each self‐reported scale showed a progressive increase (Figure 3a–e) or decrease (Figure 3c) during exercise. This suggests the scales were sensitive to changes in perceptions of fatigue for the submaximal contraction protocol. Furthermore, the greatest change was observed for the Rating of Fatigue scale. The Rating of Fatigue scale is validated to discriminate subjective sensations of fatigue from exertion during prolonged exhaustive exercise (Micklewright et al., 2017). However, based on this finding, it appears that the Rating of Fatigue scale may also be able to discriminate when performing repeated submaximal contractions. In contrast, the smallest change across the protocol was observed for the RPE scale, which quantifies the subjective sensation of exertion (Micklewright et al., 2017; Whittaker et al., 2019). It is believed that the subjective sensation of exertion is predominately of central‐originating neural signalling, but heavily modulated by feedback from the periphery (i.e., afferents within exercising muscle detecting chemical and mechanical stimuli; Amann et al., 2008; Lafargue et al., 2003). Thus, it appears the RPE scale may have an inherent insensitivity to central factors of performance fatigability during the initial stage of repeated low‐intensity contractions where the accumulation of chemical and mechanical stimuli is low.

### 4.3 Predictive models of fatigability

As changes in perceptions of fatigue occur in the presence of performance fatigue, there is a clear rationale for identifying the predictive ability of the scales to determine changes in these central factors assessed with TMS. Enhanced perceptions of fatigue, derived from VAS, have been reported to strongly correlate with the intensity and duration of MVC following sustained exercise (Leung et al., 2004; Lyons et al., 1993). However, relatively little is known on the use of other self‐reported scales as predictors of torque‐derived measures of performance fatigability during exercise. In the current study, each self‐reported scale was able to predict the torque‐derived measures assessed throughout the contraction protocol. The RPE and Rating of Fatigue scales were considered the best predictors of MVC torque. However, the prediction error of each scale was marginal between the two. Because of this, the current study is unable to distinguish which scale should be used to best represent torque‐derived measures of performance fatigue.

Previous research into the predictive importance of self‐reported scales on central factors of performance fatigability has been limited to changes in EMG RMS and the mean power frequency. The linear relationship between the commonly used RPE scale and decrease in mean power frequency of EMG is non‐existent at low loads. However, it strengthens when performing prolonged higher intensity contractions (>20% MVC; Pearson's correlation *r* = 0.46–0.79; Chan et al., 2000; Hummel et al., 2005; Kankaanpaa et al., 1997; Oberg et al., 1994). In the current study, similar relationships were found for EMG RMS assessed using the Likert and RPE scales (*r =* 0.75–0.76) when performing the contraction protocol compared to previous research into sustained submaximal contractions. In addition, we provide explanation on the underlying mechanism responsible for the reduction in EMG RMS by providing measures of excitability (MEP) and inhibition (EMG silent period) of the corticospinal pathway, and their correlative relationship with measures of perceptions of fatigue. The VAS marginally out‐performed the other subjective scales for both MEP and silent period and showed linear correlative relationships. Indeed, as the duration of the silent period increased, so too did the reported VAS score. However, like the torque‐derived measures, the prediction error associated was not sufficiently different to select one scale as being a better predictor than the others.

### 4.4 Considerations

Previous investigations have indicated that cognitive load can be a confounding factor when assessing perceptions of fatigue (Kluger et al., 2013). That is, the more cognitively demanding a task is, greater perceptions of fatigue will be reported by the individual (Blakely et al., 2022; Marcora et al., 2009; Pageaux, 2014). However, cognitive‐load can be mitigated by the application of familiarisation, which was employed in the current study the day prior to testing. Indeed, Abdel‐Malek et al. (2020) demonstrated that a 3‐day familiarisation protocol significantly reduced the discrepancy between self‐reported scales and performance fatigability measures. Furthermore, the current study only provided familiarisation of the self‐reported scales and TMS measures and not details on the number of contractions being performed. This is important to note, as prior knowledge of the 10‐phase contraction protocol may have influenced the completion of the self‐reported scales on subsequent contractions.

Sex‐based differences in the perceptions of pain have previously been reported for work‐related injuries (Cote, 2012); however, it is unclear if such differences persist for perceptions of fatigue during exercise, especially since muscle fatigue is task‐specific (Hunter, 2009). Previous assessments of RPE during biceps brachii contraction tasks have been shown to not differ between sexes when performing sustained submaximal elbow flexions (Hunter et al., 2004; Yacyshyn et al., 2018). Nonetheless, sex‐based differences in other measures of perceptions of fatigue and their relationship with neuromuscular measures of performance fatigability requires further investigation.

### 4.5 Conclusion

This study aimed to establish the extent which self‐reported scales of perceptions of fatigue can predict central factors contributing to performance fatigue. Each subjective scale studied was sensitive to the progression of performance fatigability throughout the contraction protocol. The RPE and Rating of Fatigue scales showed marginally greater predictive value for MVC torque, while the VAS was marginally the best predictor for MEP amplitude and silent period duration. Somewhat surprisingly, relative to the subjective scales, the number of contractions performed was the best predictive of VA, estimated resting twitch torque and MEP amplitude. Our findings indicate that the subjective scales of RPE, OMNI, Likert, Rating of Fatigue, and VAS may all perform similarly well when predicting neuromuscular measures of performance fatigability during sustained submaximal elbow flexions. Future studies are needed to determine whether the studied scales perform similarly in other exercise contexts, including lower limb tasks that elicit greater deficits in VA and between the sexes.

## AUTHOR CONTRIBUTIONS

Data collection was performed at the Neural Control of Movement Laboratory, Griffith University, Gold Coast, Australia. All authors contributed to the conception of this work, acquisition, analysis, and interpretation of the data, as well as the drafting of the work. All authors have approved the final version of this manuscript and agree to be accountable for all aspects of the work regarding the accuracy and integrity of the work. All persons designated as authors qualify for authorship, and all those who qualify for authorship are listed.

## CONFLICT OF INTEREST

The authors declare that no conflicts of interests exist. The results of the present study do not constitute endorsement by ACSM. The results of the study are presented clearly, honestly, and without fabrication, falsification, or inappropriate data manipulation.

## FUNDING INFORMATION

None.

## Supporting information

Statistical Summary Document

Supplementary material

## Data Availability

The data that support the findings of this study are available from the corresponding author upon reasonable request
