# Supplementary material for: Perceptions of fatigue and neuromuscular measures of performance fatigability during prolonged low‐intensity elbow flexions
Source: Exp Physiol. 2023 Feb 10;108(3):465–79. doi: 10.1113/EP090981 (PMC10103868; doi:10.1113/EP090981)

SUPPLEMENT DOCUMENTS

Supplement 1. Exploratory data analysis of fatigue outcomes for the contraction protocol. Data in the left column represent the torque-derived variables of MVC torque (A), voluntary activation (B), and resting twitch amplitude (C), presented across the 10 contraction blocks performed by participants. Data in right column represent EMG-derived variables of RMS amplitude (D), MEP/Mmax area (E), and silent period duration (F). All measurements were obtained at the completion of each 2 min submaximal contraction. The contraction block labelled 0 is the baseline unfatigued measurement for the variable. Grey lines indicate individual participant data, with the mean response indicated by the red line.

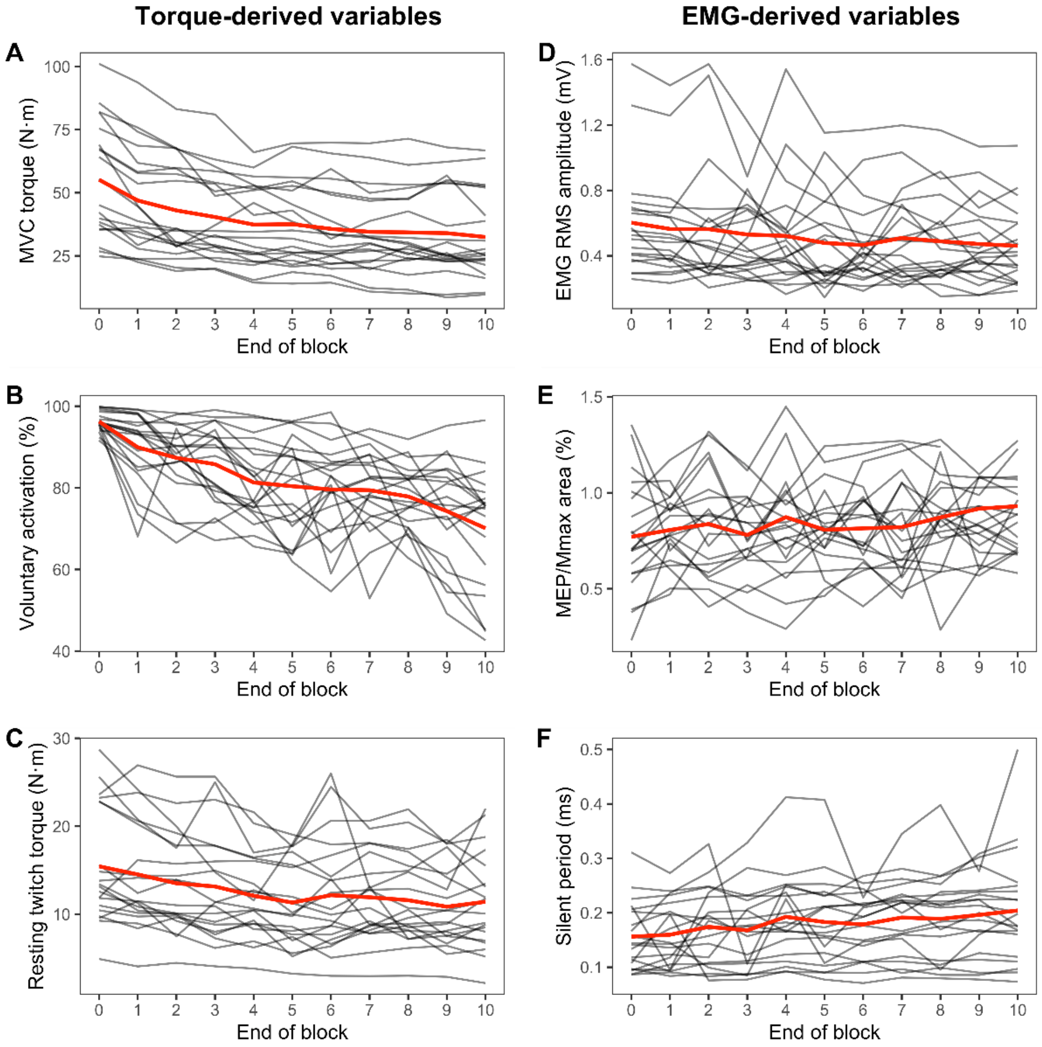

Supplement 2. Data analysis method: Model selection.

To identify the best fitting model for each outcome variable, the five models, and a base model including block, were ranked using the root-mean-square error (RMSE) statistic. The RMSE is the standard deviation of the residuals (i.e., prediction errors); with lower values indicating a better performing model. Cross validation (10-fold, with 5 repeats) was used to estimate the average RMSE for each model.[1] Using this method, the dataset was randomly split into 10 parts, with each part balanced for participants. Each model was fit on 90% of the data and tested on the remaining 10%, with the RMSE value retained and the model discarded. This process was repeated until each of the 10 parts were treated as the test dataset (and was re-run 5 times). The average RMSE was calculated from the sample of 50 RMSE values (i.e., 10 folds by 5 repeats).

We also obtained the average marginal and conditional coefficient of determination (R^2) from the cross-validation procedure.[2] The marginal R^2 reflects the variance explained by the fixed effects only, and the conditional R^2 the variance explained by both the fixed and random effects. These indices can be affected by overfitting, and as such, were not used for model selection. Information criteria (e.g., Akaike’s or Bayesian) were not used for model selection, because we didn’t want to penalise models for the number of parameters, which for the ordinal measures reflect the number of levels on a given subjective scale.[3]

Supplement 3. Boxplots of (A) RPE, (B) OMNI scale, (C) Likert scale, and (D) rating of fatigue scale responses across the contraction blocks.

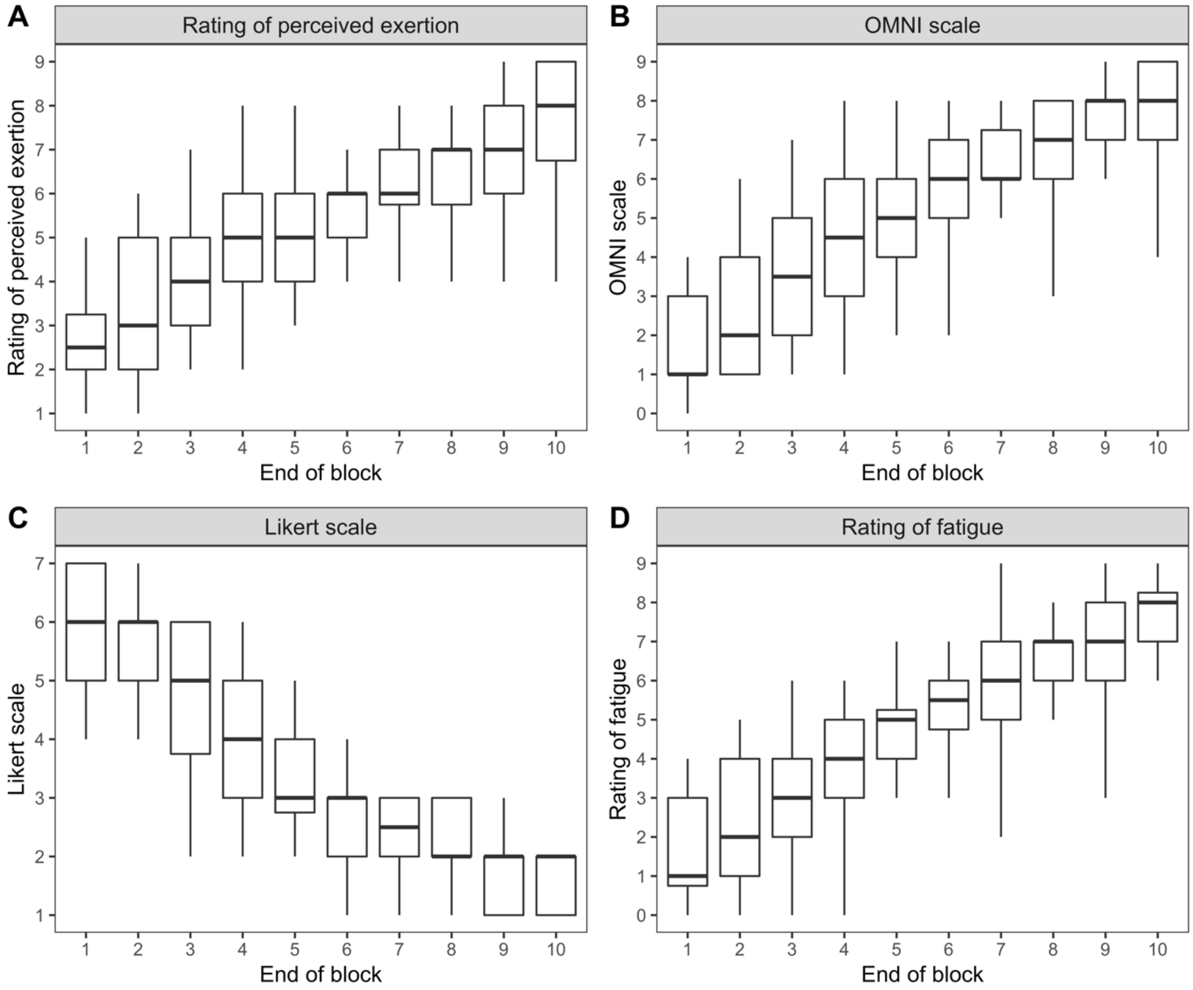

Supplement 4. Root-mean-square error (RMSE) of models predicting maximal voluntary contraction (MVC) torque (A), voluntary activation (B), resting twitch torque (C), EMG RME amplitude (D), MEP/Mmax area (E), and silent period duration (F). This includes a model where 'ratings' on a 1–10 scale were randomly generated (i.e., random), where this variable was subsequently included as a predictor. MVC torque, resting twitch torque, and EMG RMS amplitude were logged before analysis. Models are ranked according to their RMSE value, in descending order, with smaller RMSE values indicating a better performing model. Base = A base model, with contraction block included as a predictor variable, RPE = Rating of percevied exertion,VAS = Visual analogue scale.

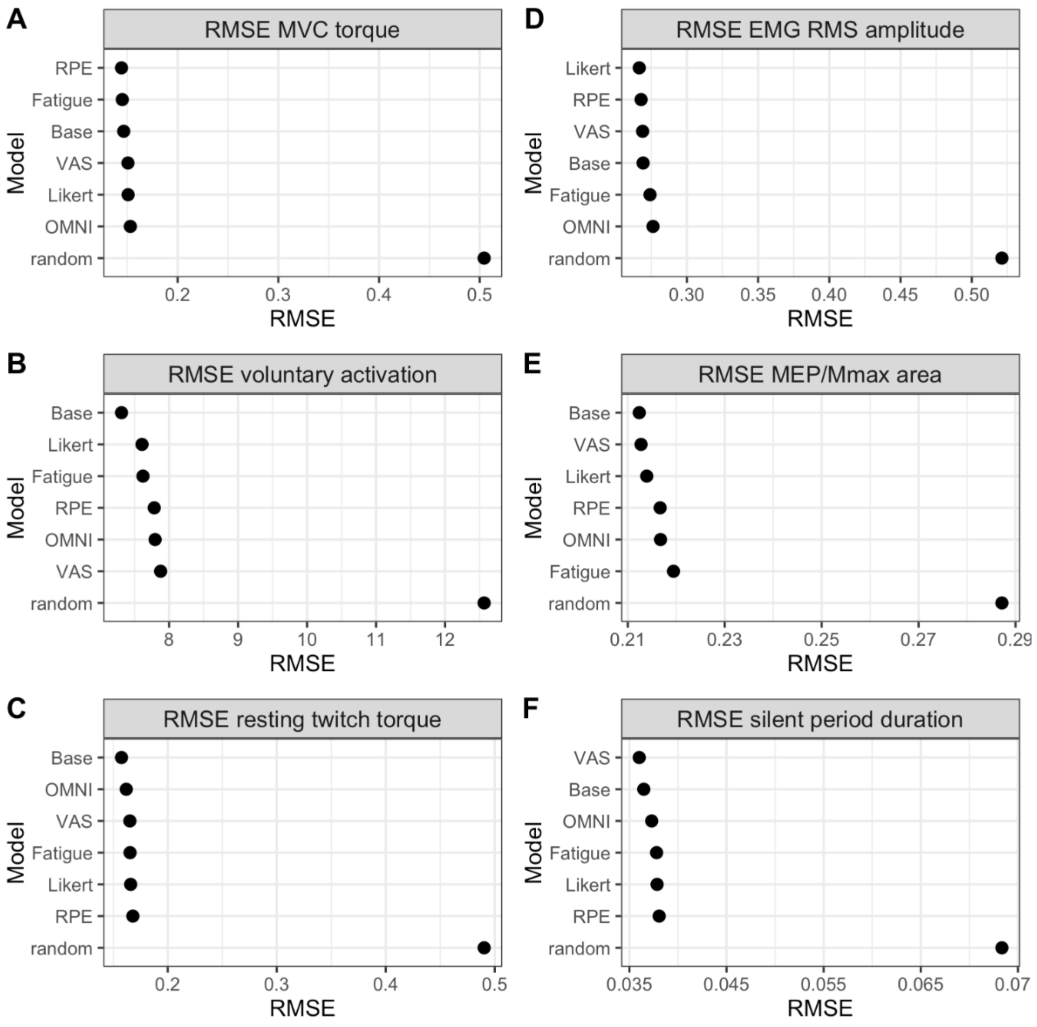

Supplement: Supplementary file 2 — Supplementary material [file EPH-108-465-s001.pdf]
